# Our understanding of the role of exercise intensity can only be precise as our classification of exercise intensity

**DOI:** 10.1113/EP091512

**Published:** 2023-09-26

**Authors:** Jayson R. Gifford, Joseph Larsen

**Affiliations:** ^1^ Brigham Young University Provo UT USA

**Keywords:** critical power, exercise intensity, exercise prescription, post‐exercise hypotension

1

The physiological responses to exercise vary with exercise intensity. Traditionally, intensity of exercise has been classified relative to maximum rate of oxygen consumption (i.e., %${\dot{V}}_{O_{2}\max}$) or maximum heart rate (i.e., %HR_max_). This traditional mode of intensity classification does provide insight into general trends, but three often unacknowledged shortcomings prevent it from having the precision needed to accurately prescribe similar cardiometabolic conditions from one person to the next.

First, it should be acknowledged that the physiological responses to exercise do not consistently change as %${\dot{V}}_{O_{2}\max}$ increases (i.e., a 10% increase in %${\dot{V}}_{O_{2}\max}$ does not necessarily equate to a 10% increase in cardiometabolic strain). As evidenced by abrupt changes in blood lactate as intensity exceeds the lactate threshold (LT) and dramatic decreases in endurance and muscle phosphocreatine levels as intensity surpasses critical power (CP) small differences in %${\dot{V}}_{O_{2}\max}$ can have large or small impacts on cardiometabolic strain depending upon how such changes relate to important physiological thresholds (Poole et al., 2016).

Second, it should be acknowledged that cardiometabolic conditions associated with exercising at a given %${\dot{V}}_{O_{2}\max}$ do not necessarily remain constant throughout an exercise. Due to growing fatigue and factors like the ${\dot{V}}_{O_{2}}$ slow component, a workload that elicited a given %${\dot{V}}_{O_{2}\max}$ or %HR_max_ during a previous test can elicit a much greater %${\dot{V}}_{O_{2}\max}$ or %HR_max_ if sustained for more than a few minutes. Even workloads associated with 50% ${\dot{V}}_{O_{2}\max}$ during a graded exercise test will elicit ${\dot{V}}_{O_{2}\max}$ or HR_max_ when sustained for more than a few minutes if that workload is above the person's CP (Poole et al., 2016).

Third, it should be acknowledged that having subjects exercise at a fixed percentage of ${\dot{V}}_{O_{2}\max}$ or HR_max_ does not guarantee that similar cardiometabolic conditions are experienced by each subject. The cardiometabolic strain of exercise pivots around physiological thresholds, like LT and CP, which do not occur at the same %${\dot{V}}_{O_{2}\max}$ or %HR_max_ for everyone (Collins et al., 2022; Meyler et al., 2023; Poole et al., 2016). Consequently, the power output associated with a fixed %${\dot{V}}_{O_{2}\max}$ (e.g., 70% ${\dot{V}}_{O_{2}\max}$) during a graded exercise test could be above CP for one person and below CP for another, which could result in wildly different levels of cardiometabolic strain for each person (Meyler et al., 2023).

Recognition of these shortcomings has led several researchers to recommend classifying exercise intensity in relation to physiological thresholds, like LT and CP (Collins et al., 2022; Meyler et al., 2023). Indeed, prescribing the intensity of exercise in relation to CP has been shown to produce more homogeneous responses to exercise (Meyler et al., 2023; Poole et al., 2016) and more predictable adaptations (Collins et al., 2022) to exercise training than traditional exercise prescription.

In this issue of *Experimental Physiology*, Lei et al. applied a threshold‐based classification system of intensity to re‐examine the role of intensity in post‐exercise hypotension. Several previous investigations (MacDonald, 2002) that described exercise intensity in relation to ${\dot{V}}_{O_{2}\max}$ reported an inconsistent relationship between intensity and the magnitude of hypotension and suggested that post‐exercise hypotension is more dependent upon total exercise volume (intensity × duration) than intensity. Of course, describing exercise by ${\dot{V}}_{O_{2}\max}$ involves the three shortcomings in precision described above, which may have obscured the true effect of exercise intensity on post‐exercise hypotension.

After determining each subject's gas exchange threshold (GET), a surrogate of LT, and CP, Lei et al. quantified post‐exercise hypotension following equal amounts (i.e., volume matched) of exercise performed above and below their personal GET and CP. With this state‐of‐the‐art categorization of exercise intensity, Lei and colleagues found that post‐exercise hypotension was markedly greater following exercise above CP (i.e., severe intensity exercise) than equal amounts of exercise below CP (i.e., heavy or moderate intensity exercise). Thus, when exercise intensity is accurately and precisely classified, it seems to have an impact on the magnitude of post‐exercise hypotension. With post‐exercise hypotension being predictive of blood pressure adaptations to exercise training (MacDonald, 2002), these results highlight the potential utility of severe‐intensity exercise in the treatment of high blood pressure.

While Lei et al. observed little‐to‐no hypotension following exercise performed below CP, it is unlikely that intensity is the only regulator of the magnitude of post‐exercise hypotension. Lei's study required subjects to perform exercise above CP until exhaustion, while exercise below CP was only performed until matching the amount of work performed in the exhaustive trial above CP. Consequently, the magnitude of cardiometabolic strain was submaximal in all trials except that above CP. It is possible that the growing cardiometabolic strain associated with prolonged exercise (e.g., ${\dot{V}}_{O_{2}}$ slow component) could elicit substantial hypotension when exercising below CP (MacDonald, 2002). Subsequent studies should determine if exhaustive exercise performed above and below CP results in differing magnitudes of post‐exercise hypotension.

In conclusion, the results of Lei et al.’s study and several other recent studies (Collins et al., 2022; Meyler et al., 2023; Poole et al., 2016) show that the seemingly random responses to traditionally prescribed exercise can often be explained, at least in part, when intensity is considered in relation to physiological thresholds. Future studies should consider classifying exercise based on physiological thresholds, like CP, rather than arbitrarily selected percentages of ${\dot{V}}_{O_{2}\max}$ or HR_max_. Ultimately, our understanding of the relationship between exercise intensity and the various responses to exercise can only be as precise as our classification of exercise intensity.

## AUTHOR CONTRIBUTIONS

Both authors approved the final version of the manuscript and agree to be accountable for all aspects of the work in ensuring that questions related to the accuracy or integrity of any part of the work are appropriately investigated and resolved. Both persons designated as authors qualify for authorship, and all those who qualify for authorship are listed.

## CONFLICT OF INTEREST

The authors declare no conflicts of interest.

## FUNDING INFORMATION

None.
